# Influence of Partial Replacement of Si by Al on Microstructure and Properties of Nanostructured Martensitic Steel

**DOI:** 10.3390/ma12223718

**Published:** 2019-11-11

**Authors:** Hua Zheng, Feng Hu, Wen Zhou, Oleg Isayev, Oleksandr Hress, Serhii Yershov, Kaiming Wu

**Affiliations:** 1 The State Key Laboratory for Refractories and Metallurgy, Wuhan University of Science and Technology, Wuhan 430081, China; 2 Collaborative Innovation Center for Advanced Steels, International Research Institute for Steel Technology, Wuhan University of Science and Technology, Wuhan 430081, China; 3 School of Nuclear Technology and Chemistry & Biology, Hubei University of Science and Technology, Xianning 437100, China

**Keywords:** nanostructured martensitic steel, quenching-partitioning-tempering, replacement of Si by Al, retained austenite

## Abstract

Nanostructured martensitic steels comprising nanoscale-martensite and retained austenite were obtained by quenching–partitioning–tempering (Q–P–T) treatment. The influence of partial replacement of Si by Al on the microstructure and properties were studied. Results showed that with partial replacement of Si by Al, except nanoscale-martensite and retained austenite, a little ferrite was also clearly observed. By contrast, with partial replacement of Si by Al, although the ultimate tensile strength (1392 MPa against 1215 MPa) was slightly lower, the elongation after fracture (16.7% against 19.9%) and the toughness (equivalent to the area under the stress–strain curve) (43.1 × 10^6^ against 37.1 × 10^6^ J/m^3^) were relatively higher. This was because the retained austenite in the steel with partial replacement of Si by Al had higher carbon content, similar volume fraction of film-like morphology, lower volume fraction of blocky morphology and was surrounded by ferrite, which played significant role in the transformation-induced plasticity (TRIP) effect.

## 1. Introduction

Recently, nanostructured martensitic steels with balanced strength and plasticity treated by quenching and partitioning (Q&P) [1,2] or quenching–partitioning–tempering (Q–P–T) [3,4] treatment have attracted attention. The processes of Q&P and Q–P–T, which were distinct from quenching and tempering (Q-T), were used for low/medium-carbon steel to generate microstructure with the nanoscale martensite (M) and retained austenite (RA) combination giving attractive properties. Usually, the element Si was added into the steel with Q&P or Q–P–T treatment to inhibit formation of carbides [5], thereby contributing to the complete diffusion of carbon to untransformed austenite from supersaturated-carbon martensite, thus increasing stability of RA upon subsequent cooling to ambient temperature. Besides, the strong solid solution strengthening of Si can enhance the strength of the steel. However, high content of Si in steel can lead to the problems of surface quality and coating performance [6], and also result in poor weldability.

Al, similar to Si, can strongly prevent the precipitation of cementite and adjust the stacking fault energy of RA [7]. Recently many researchers have tried to use Al to completely or partially replace Si in steels [8,9]. Some previous studies have shown that the addition of Al accelerates the pearlitic [10] and/or bainitic [11] transformation, therefore shortening the transformation time of austenite to pearlite and/or bainite. In transformation-induced plasticity (TRIP) steel, with partial replacement of Si by Al, the ferritic transformation was accelerated during the subsequent heat-treatment after intercritical annealing [8], which can be attributed to the increase of free energy during the transformation of austenite to ferrite. Both the low-temperature bainite steels and TRIP steels contain some retained austenite. Compared with them, the martensite steels treated with Q–P–T process also contain retained austenite. The partial replacement of Si by Al may be an important impact on the microstructure of the Q–P–T-treated martensite steels, especially on the retained austenite, and further affect its mechanical properties. However, at present, the influence of Al on the transformation of martensite is not well understood for the martensite steels. In this study, we not only examine phase transformation and mechanical properties of the Q–P–T-treated steels, but also investigate the influence of partial replacement of Si by Al for Q–P–T-treated martensite steels.

## 2. Materials and Methods

In this study, two experimental steels were prepared by 50 kg vacuum melting in a medium frequency furnace and were casted into ingots. The ingots were reheated to 1200 °C and hot-rolled to 20 mm thickness. The chemical composition of the investigated steels in wt% were Fe-0.43C-1.98Mn-1.54Si-0.38Al (referred to as 1.54Si) and Fe-0.44C-1.94Mn-0.58Si-1.26Al (referred to as 1.26Al), with an appropriate amount of Ti addition. The amount of Si + Al in the investigated steels remained approximately constant, while the ratio of Si to Al was changed (4:1 and 1:2, respectively). The addition of medium-carbon was for high strength, Mn was for improving hardenability, Si and Al were for preventing the precipitation of cementite, and Ti was for refining the grain. After the chemical composition was determined, the MUCG83 thermodynamic model [12] developed by Bhadeshia was used to estimate the phase transition temperature and calculate the time–temperature–transformation (TTT) curves, which were useful for planning the heat treatment process.

Specimens were austenitized at 1000 °C for 30 min and then were quenched in a molten salt-bath at 210 °C for 2 min. This was followed with partitioning-tempering at 450 °C for different periods in a molten salt-bath, and finally quenched to room-temperature water.

The metallographic specimens for scanning electron microscopy (SEM), using a Sirion 200, were ground, mechanically polished, and etched with 4 vol.% Nital solution. The morphology and orientation of the martensite laths were characterized by electron back-scatter diffraction (EBSD, JEOL-7600F, JEOL, Tokyo, Japan). The EBSD samples underwent standard mechanical polishing to 0.05 μm, followed by electropolishing in a 6% perchloric acid and 94% acetic acid solution (by volume). The details of the working conditions for EBSD analyses were as following: accelerating voltage of 15 kV, working distance of 15 mm, tilt angle of 70°, and step size of 100 nm.

Specimens for transmission electron microscopy (TEM), using a JEM-2010HT (JEOL, Tokyo, Japan), were machined from 3 mm diameter rods which were sliced into 100 mm discs. These thin foils were ground down to 50 µm thickness, and then electropolished at 50 V using a twin-jet unit using an electrolyte solution of 5% perchloric acid, 15% glycerol, and 80% methanol. Meanwhile, thickness of martensite laths (t) was determined by the mean lineal intercept (L = π t/2) using TEM with at least 10 micrographs [13].

X-ray diffractometry (XRD) with Cu Kα radiation was used to step-scan the specimens, and the 2θ scan angles ranged from 30° to 100°. With the measurement error of 1.5 vol%, the volume fraction of RA was determined by calculating the mean integrated intensities of the (111), (200), (220) and (311) austenite peaks and (110), (200), (211) and (202) martensite peaks [14]. The carbon content in RA was estimated with the measured lattice parameters, and can be calculated by the following expression [15,16]. The calculation error was about 0.10 wt%.

α_γ_ = 3.5780 + 0.033C_γ_ (1)

where α_γ_ is the lattice parameter of austenite in Å, and C_γ_ is the carbon concentration of austenite.

Electron probe microanalysis (EPMA) was used to analyze the line distribution of carbon and the carbon content for different morphologies of RA in specimens.

Each value of Vickers hardness represented an average of ten measurements (1.0 kg load). For tensile testing, there were three specimens for each heat treatment process and the reported values were the average of three measurements. According to the GB/T 228.1-2010 standards, the tensile test specimens were cut to the length of 100.0 mm, gauge length of 31.0 mm, gauge width of 10.0 mm and thickness of 3.0 mm, and performed on a Zwick T1-FR020TN A50 tensile-testing machine (Zwick, Ulm, Germany) with the strain rate of 10^−3^ s^−1^ at room temperature.

## 3. Results

### 3.1. Microstructure Analysis

**Figure 1:**
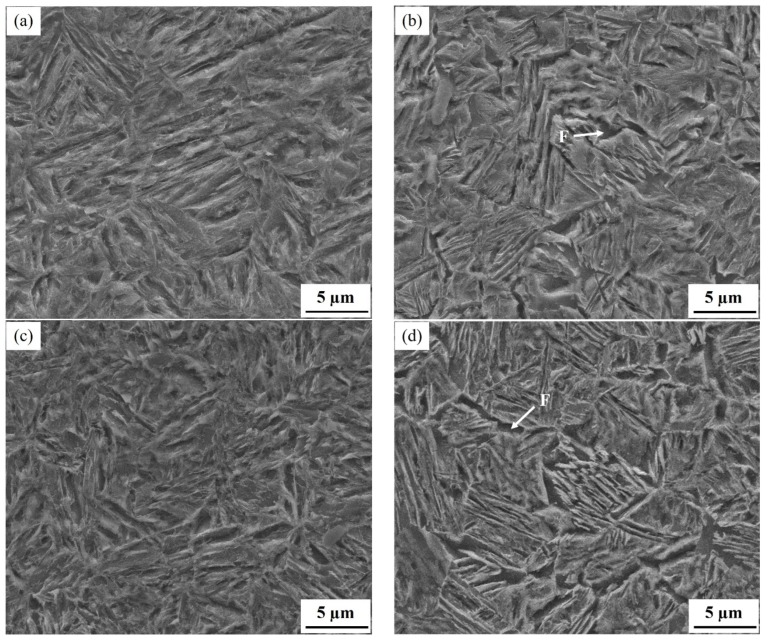

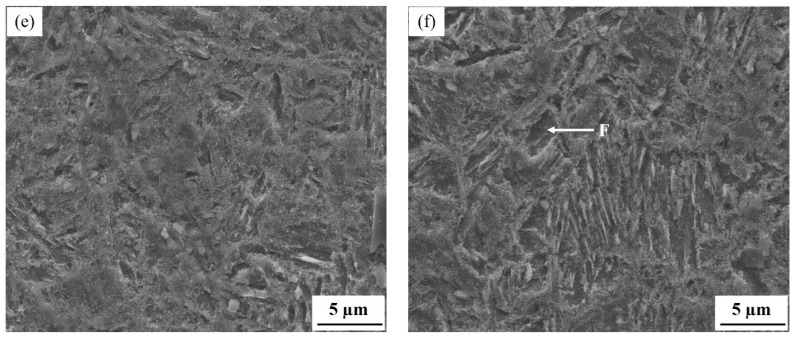
SEM micrographs of specimens of 1.54Si steel (**a**,**c**,**e**), and of 1.26Al steel (**b**,**d**,**f**): (**a**,**b**) as-quenched at 210 °C; (**c**,**d**) quenched at 210 °C and partitioned-tempered at 450 °C for 60 s; (**e**,**f**) quenched at 210 °C and partitioned-tempered at 450 °C for 1800 s.

Figure 1 shows typical SEM micrographs for the specimen as-quenched at 210 °C and partitioned-tempered at 450 °C for 60 s and 1800 s, which consist of typical martensite (M) and retained austenite (RA) dispersedly distributed in the matrix in both alloys. However, a visible amount of ferrite (F) was also observed in the steel of 1.26Al. Compared with the specimen as-quenched, with the increase of partitioned-tempered time (60 s), the volume fraction of RA was decreased. When the partitioned-tempered time was prolonged to 1800 s, a lot of fine carbides precipitated from the microstructure of martensite and retained austenite, and the martensite laths became coarsened.

**Figure 2:**
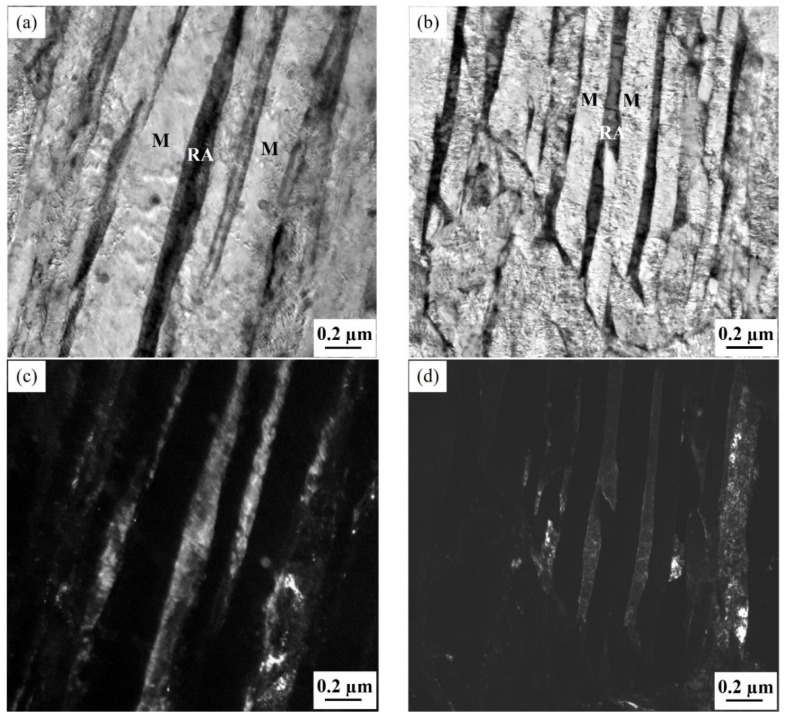

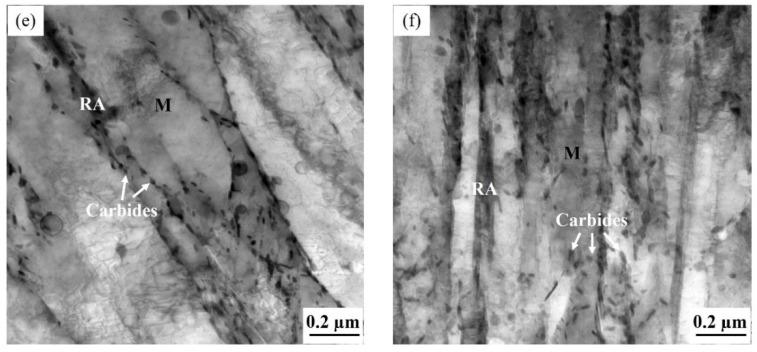
TEM micrographs of the specimens: (**a**,**c**) for the bright and dark field image of 1.54Si-60s; (**b**,**d**) for the bright and dark field image of 1.26Al-60s; (**e**,**f**) for the 1.54Si-1800s and 1.26Al-1800s, respectively.

Figure 2 shows typical TEM micrographs with nanoscale-martensite and retained austenite for the specimens partitioned-tempered at 450 °C for 60 s and 1800 s. The thickness of the nanoscale-martensite lath for the specimen of 1.54Si partitioned-tempered for 60 s was 206 ± 39 nm, while the thickness for the steel of 1.26Al was 102 ± 20 nm. With the partial replacement of Si by Al, the martensite laths became fine (~100 nm). When the partitioned-tempered time was prolonged to 1800 s, the martensite laths became coarsened, the interface became unclear, and a lot of carbides precipitated from the martensite laths.

**Figure 3:**
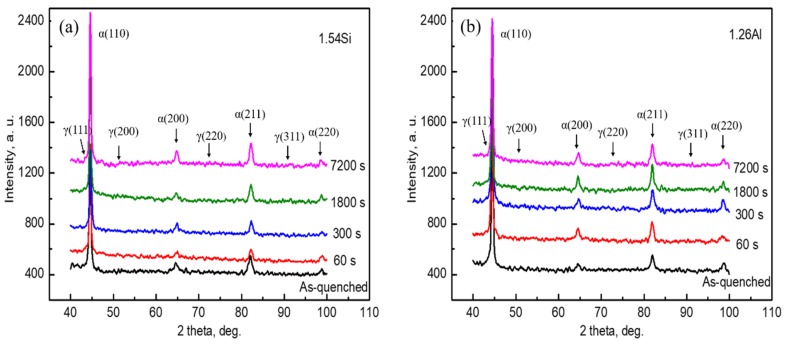

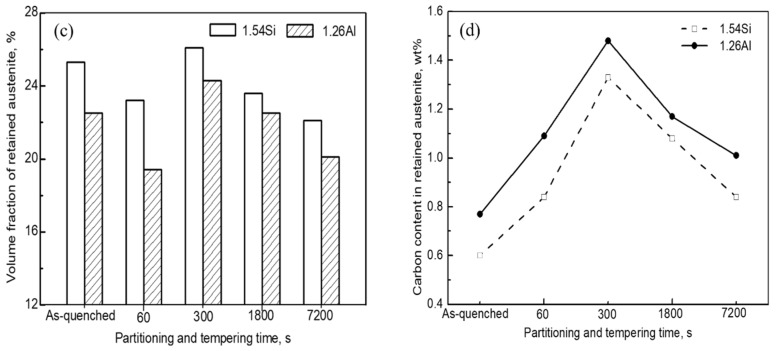
XRD results for the specimens with Q–P–T (Quenching–Partitioning–Tempering) treatment: (**a**) XRD pattern for the 1.54Si steel; (**b**) XRD pattern for the 1.26Al steel; (**c**) the volume fraction of RA (Retained Austenite) in different specimens; (**d**) the carbon content of RA in different specimens.

Figure 3 shows the XRD results for the specimens with Q–P–T treatment. Figure 3a,b exhibits the XRD pattern for the 1.54Si and 1.26Al steels, respectively. Figure 3c shows the volume fraction of RA for the different specimens with Q–P–T treatment. It can be seen that with the addition of 1.26Al, the volume fraction of RA was relatively lower than that of the 1.54Si steel. The volume fraction of RA for the specimens as-quenched at 210 °C were 25.3 vol% and 22.5 vol% for the 1.54Si and 1.26Al steels, respectively. Further, for the two alloys, the maximum RA fractions of 26.1 and 24.3 vol% were obtained when partitioned-tempered at 450 °C for 300 s. With the partitioned-tempered time increasing (7200 s), the amount of austenite was decreased (22.1 vol% and 20.1 vol% were obtained for the 1.54Si and 1.26Al steels, respectively) (carbides were not taken into account). Figure 3d shows the carbon content of RA for the specimens with Q–P–T treatment. It can be seen that partial-replacement of Si by Al in steel increased its carbon content. The maximum level of carbon content of RA (1.33 wt% and 1.48 wt% in the 1.54Si and 1.26Al steels quenched at 210 °C, respectively) was obtained for specimens partitioned-tempered at 450 °C for 300 s. With the partitioned-tempered time increasing (7200 s), less carbon enrichment in austenite (0.84 wt% and 1.01 wt% for the 1.54Si and 1.26Al steels, respectively) was observed.

**Figure 4:**
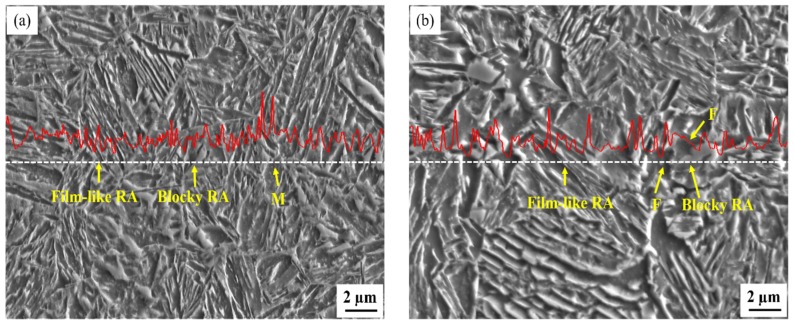
The line distribution of carbon by EPMA (Electron Probe Microanalysis) of samples partitioned-tempered for 60 s: (**a**) for 1.54Si steel and (**b**) for 1.26Al steel.

Figure 4 shows the line distribution of carbon by EPMA for the 1.54Si-60s and 1.26Al-60s specimens. The dotted line with white color was the scanning route we selected, and the red curve was the line intensity which related to the carbon content. The carbon content in different phases was not the same. As the scanning line passed through different phases, the intensity line fluctuated with changing carbon content. It was clearly exhibited that carbon was distributed differently in different phases and even in different morphologies of RA. As shown in Figure 4, the morphologies of RA for the 1.54Si and 1.26Al steels are film and block. In the steel of 1.54Si, RA was distributed around the martensite laths with film and block morphologies. In the 1.26Al steel, except around the martensite laths with film and block morphologies, some austenite was surrounded with ferrite.

**Figure 5:**
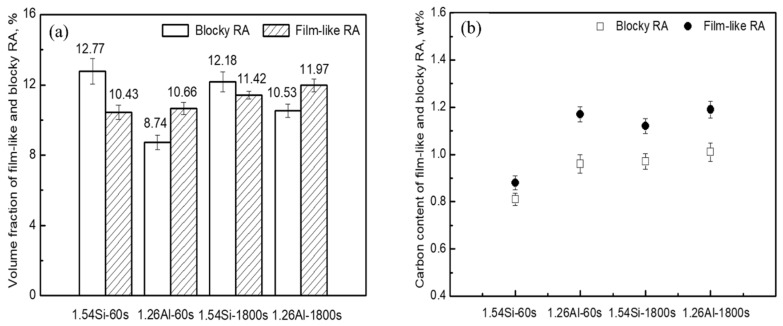
(**a**) the volume fraction of blocky and film-like RA; (**b**) the carbon content of blocky and film-like RA in different heat-treated specimens.

The volume fraction and carbon content of blocky and film-like RA in differently heat-treated specimens are shown in Figure 5. The rough calculation of volume fraction for RA with different morphologies was done using Image-Pro software. Figure 5a indicated that in the 1.54Si steel, the total volume fraction of RA was relatively higher, while there was mainly blocky RA. Corresponding, in the steel of 1.26Al, the total volume fraction of RA was relatively lower, while there was mainly film-like RA. Simultaneously, compared with the 1.54Si steel, the volume fraction of film-like RA was similar, but the volume fraction of blocky RA was significantly reduced in the 1.26Al steel. Figure 5b indicated that the carbon content of film-like RA was higher than that of blocky RA for all the specimens, and the partial replacement of Si by Al in steel leads to the increase in the carbon content of blocky and film-like RA.

**Figure 6:**
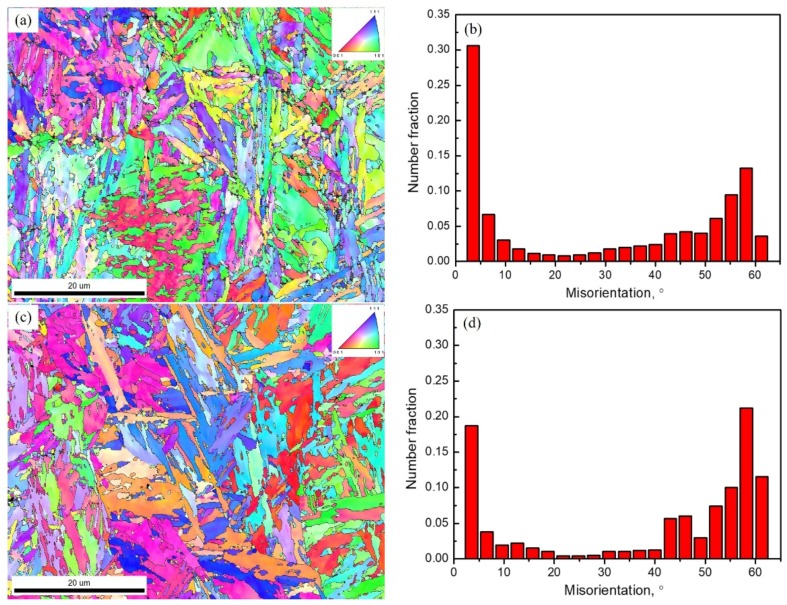
EBSD microstructure characterization for the sample of 1.54Si-60s (**a**,**b**), the sample of 1.26Al-60 s (**c**,**d**): Orientation image (left) and misorientation angle distribution (right).

Figure 6 shows the EBSD microstructure characterization for the samples. Both the 1.54Si and 1.26Al samples had a larger proportion of small grain boundary misorientation angles (<15°), while the large grain boundary misorientation angle (>15°) was 57.9% and 73.3% for the 1.54Si and 1.26Al samples, respectively. They both exhibited peaks in misorientation angles in the 3–5° and 55–60° number fractions. By comparison, for the sample of 1.26Al, the 3–5° number fraction peak exhibited an evident reduction while the 55–60° number fraction peak exhibited an evident increase.

### 3.2. Mechanical Properties

**Figure 7:**
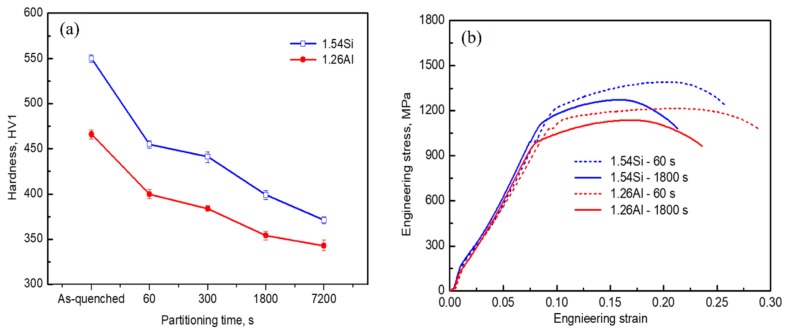
Mechanical properties of the samples: (**a**) hardness; (**b**) engineering stress–strain curves.

Figure 7a shows the measured hardness for the specimens with Q–P–T treatment. The maximum hardness values of 550 and 466 HV1 were obtained for the steel of 1.54Si and 1.26Al as-quenched at 210 °C, respectively. With the increase of partitioning-tempering time (60 s), the hardness was decreased and remained about 455 and 400 HV1 for these two alloys. When the partitioning-tempering time was further increased up to 7200 s, the hardness dropped sharply (371 and 343 HV1, respectively) due to the coarsening of martensite plates and precipitated carbides.

The engineering stress–strain curves are shown in Figure 7b, and the data of corresponding properties are displayed in Table 1. In particular, the elongation after fracture (A) was calculated with the ratio of the total deformation length for the gauge section after tensile fracture to the original gauge length, and the area under the stress–strain curve (ASSC) was used as a measure of the toughness for the specimens. It can be seen that partial-replacement of Si by Al in steel decreases the ultimate tensile strength (UTS), while increases the elongation after fracture (A) and the toughness (area under the true stress–strain curve). For instance, the 1.26Al steel with relatively smaller ultimate tensile strength (i.e., 1215 MPa compared to 1392 MPa with the same partitioning-tempering time of 60 s) had higher elongation after fracture (i.e., 19.9% as to 16.7%) and much more area under the true stress–strain curve (i.e., 43.1 × 10^6^ J/m^3^ compared to 37.1 × 10^6^ J/m^3^) than those of the 1.54Si steel. A longer partitioning-tempering time (i.e., 1800 s) reduced the UTS (1272 MPa and 1137 MPa, respectively) and elongation after fracture (12.3% and 14.6%, respectively) in both steels. At a short partitioning-tempering time (60 s), the toughness (area under the true stress–strain curve) for the 1.54Si and 1.26Al steels (37.1 × 10^6^ and 43.1 × 10^6^ J/m^3^, respectively) were higher, compared with the long partitioning-tempering time of 1800 s (19.4 × 10^6^ J/m^3^ and 25.7 × 10^6^ J/m^3^ for the 1.54Si and 1.26Al steels, respectively).

## 4. Discussion

### 4.1. Transformation Kinetics

**Figure 8:**
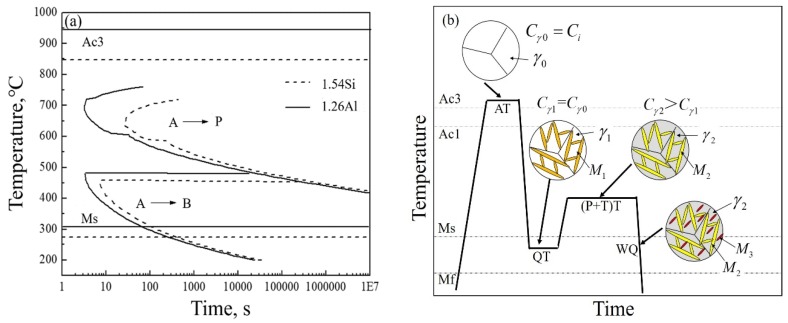
(**a**) the transformation curves of TTT (Time–Temperature–Transformation); (**b**) the schematic diagram of Q–P–T transformation. (Note: A→P—austenite transformed to pearlite, A→B—austenite transformed to bainite, A_c3_—temperature of ferrite completely transformed into austenite during heating, Ac1—temperature of ferrite start transformed into austenite during heating, M_s_—starting transformation temperature of martensite, M_f_—finishing transformation temperature of martensite, AT—austenitizing temperature, QT—quenching temperature, (P + T)T—partitioning-tempering temperature, WQ—water quenching, γ_0_—austenite in austenitizing process, γ_1_—austenite in quenching process, γ_2_—austenite in partitioning-tempering process, M_1_—martensite in quenching process, M_2_—martensite in partitioning-tempering process, M_3_—martensite in water quenching process, C_i_—carbon content of alloy elements, C_γ0_—carbon content of austenite in austenitizing process, C_γ1_—carbon content of austenite in quenching process, C_γ2_—carbon content of austenite in partitioning-tempering process).

Figure 8a exhibited the calculated time–temperature–transformation (TTT) curves for the investigated steels with MUCG 83 Mod software. It was seen that Al can raise the transformation temperature and shift the C-curve to the upper region. It meant that partial-replacement of Si by Al in steel raised the M_s_ temperature to decrease the volume fraction of RA during the subsequent cooling process. Thus, the volume fraction of RA in the 1.26Al steel was relatively lower than that of the 1.54Si steel, and this was consistent with the result shown in Figure 3a. On the other hand, owing to the high-content addition of ferrite forming element Al, the transformation temperature (A_c3_) was significantly improved and the ferrite phase was expanded [17], thus it was easy to form ferrite. The presence of ferrite resulted in carbon rejection and partitioning into adjacent austenite [18], so the 1.26Al steel was with relatively higher carbon-content in RA than that of the 1.54Si steel.

Figure 8b demonstrates the Q–P–T transformation for the 1.54Si steel and 1.26Al steel. The microstructure for the Q–P–T steels consisted of martensite, RA and carbides. After austenitizing, the carbon content in austenite was equal to that in the matrix steel (C_γ0_ = C_i_). In the subsequent quenching process, part of the austenite transformed into martensite (M_1_) and some remained (γ_1_). During the partitioning-tempering process, the microstructure was composed of tempered martensite (M_2_) and retained austenite (γ_2_). Further, there was a redistribution of some carbon from martensite to RA [19], thus the carbon content in RA of partitioning-tempering process was higher than that of quenching process for the 1.54Si and 1.26Al steels (C_γ2_ > C_γ1_). However, with partitioning-tempering time increasing (>300 s), less carbon-enrichment in austenite was observed **(**Figure 3b), possibly due to competing processes, such as carbide formation or carbon segregation to dislocations in martensite during the long partitioning-tempering time [5]. During the final quenching process, some retained austenite transformed into fresh quenched martensite (M_3_) and others remained to room temperature.

### 4.2. TRIP Effect of Retained Austenite

Retained austenite plays a crucial role in the improvement of mechanical properties for high-strength steels due to the transformation-induced plasticity (TRIP) effect [20]. The TRIP effect is attributed to the martensite transformation from metastable RA during deformation [21], thus leading to additional elongation by delaying necking [22,23]. This meant that the TRIP effect was not only related to the volume fraction of RA but also related to the stability of RA. The stability of RA was affected by many factors, such as the carbon content of RA [24], the size and morphology of RA [25], and the neighboring phase of RA [26].

Carbon is a potent stabilizer for RA by affecting the chemical driving force of martensite transformation [25]. RA with low-carbon content quickly transformed to martensite under a small amount of stress, while there was no TRIP effect in this case and it did not benefit to the increase of elogation [27]. When with appropriate high-carbon content, RA under stress transformed to martensite and the transformation plasticity produced. It effectively alleviated the local stress concentration and delayed the formation of cracks, which improved the elongation of the steels. The 1.26Al steel has the higher carbon content of retained austenite than that of 1.54Si steel. Compared with the 1.54Si steel, RA with relatively higher carbon content in the 1.26Al steel was more stable, which was a benefit of the TRIP effect, thus improving the elongation after fracture of the steel.

The size and morphology of RA have an important effect on TRIP effect. The blocky RA with large size has fewer nucleation sites for martensite transformation. It was necessary to provide greater driving force for the nucleation of martensite [28], thus, it did not benefit from the TRIP effect. On the other hand, the film-like RA with small size had more nucleation sites, and it was easier to transform to martensite. Although the total volume fraction of RA in the 1.26Al steel was relatively lower, there was mainly film-like RA and the volume fraction of film-like RA was similar to that in the 1.54Si steel. Meanwhile, although the total volume fraction of RA in the 1.54Si steel was relatively higher, there was mainly blocky RA and the carbon content of blocky and film-like RA were relatively lower. Thus, the 1.26Al steel, with an appropriate volume fraction of film-like RA and higher carbon content, exhibited an obvious TRIP effect, which was beneficial, improving in elongation after fracture of the steel.

Besides, the TRIP effect also depends on the surrounding phase of RA. In addition to RA around the martensite, there was a small fraction of RA surrounded with ferrite in the 1.26Al steel. During deformation, due to the small strain for ferrite, RA around ferrite in the 1.26Al steel experienced more stress–strain and was more prone to undergoing martensite transformation [29]. This meant that the TRIP effect in the steel of 1.26Al was more obvious than in the 1.54Si steel, thus the 1.26Al steel was given an obvious improvement in elongation after fracture.

## 5. Conclusions

Microstructure and properties for the steels of 1.54Si and 1.26Al with Q–P–T treatment were comparatively studied. Conclusions were drawn as follows:

(1) For the 1.54Si steel, multi-phase microstructure composed of nanoscale-martensite and retained austenite was obtained. With partial replacement of Si by Al, except martensite and retained austenite, a visible amount of ferrite was also observed. Meanwhile, the large grain boundary misorientation angle (>15°) in the 1.26Al steel was relatively more than that in the 1.54Si steel.

(2) With partial replacement of Si by Al, the hardness and tensile strength of 1.26Al steel were relatively lower. However, RA with higher carbon content, a similar volume fraction of film-like morphology, a lower volume fraction of blocky morphology, and ferrite surroundings, played a significant role in the TRIP effect, which contributed to the higher elongation after fracture and higher toughness (equivalent to the area under the stress–strain curve), compared to the 1.54Si steel.

## Figures and Tables

**Table 1:** Tensile properties of the investigated steel.

Specimen	P + T Time, s	UTS, MPa	A, %	ASSC, ×10^6^ J/m^3^
1.54Si	60	1392 ± 14	16.7 ± 1.3	37.1 ± 1.5
	1800	1272 ± 11	12.3 ± 0.9	19.4 ± 1.3
1.26Al	60	1215 ± 13	19.9 ± 1.2	43.1 ± 2.1
	1800	1137 ± 17	14.6 ± 0.6	25.7 ± 1.9

Note: P + T time: partitioned-tempered time; UTS: ultimate tensile strength; A: elongation after fracture; ASSC: the area under the stress–strain curve.
